# Multidisciplinary assessment of the Abbott BinaxNOW SARS-CoV-2 point-of-care antigen test in the context of emerging viral variants and self-administration

**DOI:** 10.1038/s41598-021-94055-1

**Published:** 2021-07-16

**Authors:** Jennifer K. Frediani, Joshua M. Levy, Anuradha Rao, Leda Bassit, Janet Figueroa, Miriam B. Vos, Anna Wood, Robert Jerris, Van Leung-Pineda, Mark D. Gonzalez, Beverly B. Rogers, Maud Mavigner, Raymond F. Schinazi, Nils Schoof, Jesse J. Waggoner, Russell R. Kempker, Paulina A. Rebolledo, Jared W. O’Neal, Cheryl Stone, Ann Chahroudi, Claudia R. Morris, Allie Suessmith, Julie Sullivan, Sarah Farmer, Amanda Foster, John D. Roback, Thanuja Ramachandra, CaDeidre Washington, Kristie Le, Maria C. Cordero, Annette Esper, Eric J. Nehl, Yun F. Wang, Erika A. Tyburski, Greg S. Martin, Wilbur A. Lam

**Affiliations:** 1 Nell Hodgson Woodruff School of Nursing, Emory University, Atlanta, Georgia; 2 The Atlanta Center for Microsystems-Engineered Point-of-Care Technologies, Atlanta, Georgia; 3 Department of Otolaryngology-Head and Neck Surgery, Emory University School of Medicine, Atlanta, Georgia; 4 Department of Pediatrics, Emory University School of Medicine, Atlanta, Georgia; 5 Laboratory of Biochemical Pharmacology, Emory University, Atlanta, Georgia; 6 Children’s Healthcare of Atlanta, Atlanta, Georgia; 7 Department of Pathology and Laboratory Medicine, Emory University School of Medicine, Atlanta, Georgia; 8 Department of Medicine, Emory University School of Medicine, Atlanta, Georgia; 9 Rollins School of Public Health, Emory University, Atlanta, Georgia; 10 Georgia Institute of Technology, Atlanta, Georgia; 11 Aflac Cancer and Blood Disorders Center at Children’s Healthcare of Atlanta, Emory University School of Medicine, Atlanta, Georgia; 12 Wallace H. Coulter Department of Biomedical Engineering, Emory University and Georgia Institute of Technology, Atlanta, Georgia

**Keywords:** Infectious diseases, Viral infection

## Abstract

While there has been significant progress in the development of rapid COVID-19 diagnostics, as the pandemic unfolds, new challenges have emerged, including whether these technologies can reliably detect the more infectious variants of concern and be viably deployed in non-clinical settings as “self-tests”. Multidisciplinary evaluation of the Abbott BinaxNOW COVID-19 Ag Card (BinaxNOW, a widely used rapid antigen test, included limit of detection, variant detection, test performance across different age-groups, and usability with self/caregiver-administration. While BinaxNOW detected the highly infectious variants, B.1.1.7 (Alpha) first identified in the UK, B.1.351 (Beta) first identified in South Africa, P.1 (Gamma) first identified in Brazil, B.1.617.2 (Delta) first identified in India and B.1.2, a non-VOC, test sensitivity decreased with decreasing viral loads. Moreover, BinaxNOW sensitivity trended lower when devices were performed by patients/caregivers themselves compared to trained clinical staff, despite universally high usability assessments following self/caregiver-administration among different age groups. Overall, these data indicate that while BinaxNOW accurately detects the new viral variants, as rapid COVID-19 tests enter the home, their already lower sensitivities compared to RT-PCR may decrease even more due to user error.

## Introduction

The development and expansion of current testing options remain an essential focus of ongoing efforts to confront the Coronavirus Disease 2019 (COVID-19) pandemic. Amplification-based, reverse-transcriptase polymerase chain reaction (RT-PCR) tests represent the most sensitive option for detecting severe acute respiratory syndrome coronavirus 2 (SARS-CoV-2) RNA and are recognized as the gold standard for the diagnosis of COVID-19. However, RT-PCR-based assays are not ideal or practical for all testing scenarios as the associated equipment and cost decrease their utility for rapid screening, especially in the home environment. Lateral flow antigen-detection assays (LFAs) have received great interest as an alternative testing option for the diagnosis of COVID-19^1^. Similar LFA based tests have been approved for the diagnosis of other transmissible respiratory illnesses, including influenza A/B and respiratory syncytial virus^2, 3^. Potential advantages of such assays compared to RT-PCR testing include a lower cost, rapid turnaround time, no need for specialized equipment (i.e., thermocyclers) or laboratory-based staff, and the potential for at-home use.

Despite the advantages of LFAs, the disadvantages need careful consideration. The sensitivity of SARS-CoV-2 LFA diagnostics are heterogeneous when conducted by trained healthcare workers^4^. The sensitivity of these approved LFAs have not been evaluated in untrained users. If sensitivity decreases further with untrained users, the usability of these rapid, inexpensive diagnostics lose their value. With this limited sensitivity, the ability to detect emerging virulent SARS-CoV-2 variants become concerning. It is unknown if the LFAs currently on the market are sensitive enough to detect the new, widespread variants. Low sensitivity is especially important in asymptomatic or low viral load populations^5^.

Among commercially available LFAs for the diagnosis of COVID-19, the Abbott BinaxNOW COVID-19 Ag Card (Abbott Laboratories, Abbott Park, IL), hereafter referred to as BinaxNOW, was the first LFA to receive a FDA Emergency Use Authorization (EUA) for the home setting when prescribed by a physician and, as of March 31, 2021, also authorized for over-the-counter use^6, 7^. BinaxNOW is a qualitative, SARS-CoV-2 diagnostic assay that detects the SARS-CoV-2 viral nucleocapsid (N) protein from collected anterior nasal swabs^8^. Test results are determined in 15 min via a color-based, visual indicator and no specialized equipment is needed. BinaxNOW is indicated for individuals suspected of COVID-19 by their healthcare provider within the first 7 days of symptom onset. Following the original EUA approval, the U.S. Department of Health and Human Services acquired 150 million test kits for distribution to COVID-19 testing centers, nursing homes and other points of care throughout the country^9^.

Despite the need for additional testing options and the acceptance and subsequent approval of this LFA for over-the-counter (OTC) COVID-19 diagnosis, additional field studies are critical. External studies of BinaxNOW performance in real world settings found varying results in test performance when used by trained medical personnel, and have yet to evaluate test sensitivity following self-collection or ability to detect emerging variants of concern (VOC)^10, 11^. We sought to determine (1) the BinaxNOW limit of detection (LOD) for several SARS-CoV-2 isolates, (2) its ability to detect VOC-202012/01 or B.1.1.7 (Alpha) first identified in the UK, B.1.351 (Beta) first identified in South Africa, P.1 (Gamma) first identified in Brazil, B.1.617.2 (Delta) first identified in India and B.1.2 present in the current US population at 0.1%, but considered a non-VOC. (Pango lineages https://cov-lineages.org/), (3) the performance of BinaxNOW in adult and children, and (4) complete usability assessments for patient/caregiver collection.

## Results

### Analytical sensitivity

The limit of detection (LOD) of BinaxNOW test was evaluated by using serial dilutions of live SARS-CoV-2 isolates of known concentrations. Four isolates were used including the USA-WA1/2020, USA-CA3/2020 and Italy-INMI1 isolates, as well as a new local clinical isolate we named USA-GA4/2020. The LOD was defined as the lowest virus concentration that was detected 95% of the time. BinaxNOW LOD was determined to be between 94 and 750 TCID_50_/swab (2.98 × 10^3^–7.12 × 10^4^ RNA copies/swab). See Table 1.

**Table 1:** SARS-CoV-2 live virus limit of detection.

Viral dilution tested		Positive results/replicates			
TCID_50_/ml	TCID_50_/Swab	USA-WA1/2020	USA-CA3/2020	Italy-INMI1	USA-GA4/2020
7.5 × 10^5^	1.5 × 10^4^	3/3	n.t	n.t	n.t
7.5 × 10^4^	1.5 × 10^3^	5/5	3/3	3/3	3/3
3.8 × 10^4^	7.5 × 10^2^	5/5	3/3	3/3	3/3
1.9 × 10^4^	3.6 × 10^2^	0/5	3/3	2/3	3/3
9.4 × 10^3^	1.8 × 10^2^	n.t	3/3	0/3	2/3
4.7 × 10^3^	9.4 × 10^1^	n.t	3/3	n.t	0/3
2.3 × 10^3^	4.5 × 10^1^	n.t	0/3	n.t	n.t

*TCID*_*50*_ 50% tissue culture infectivity dose, *n.t*. not tested.

### Variant testing

Panels prepared from remnant clinical samples were used to evaluate BinaxNOW. Results using pooled panels prepared from remnant clinical samples show that BinaxNOW detects B.1.1.7 CoV-2 Ag at cycle threshold (Ct) values up to 27 (for CDC-N2 gene). A side-by-side comparison with B.1.2, a non-VOC, showed detection at a similar range. In addition, BinaxNOW also detected two other VOCs including P.1, B.1.351 with the latest having high Ct values varying of ~ 26, when using non-inactivated remnant clinical samples (Table 2).

**Table 2:** Sensitivity of BinaxNOW for four relevant VOC versus Cycle threshold (Ct) values by RT-PCR.

	Ct range of CDC N2 gene (BinaxNOW—positive/total)			
	17–20	20–22	24–27	29–35
B.1.1.7^a^	7/7	6/6	6/6	0/6
B.1.2^a^	7/7	6/6	6/6	0/6
B.1.1.7^b^	5/5	nd	nd	nd
B.1.2^b^	6/6	nd	nd	nd
B.1.351^b^	nd	nd	2/2	nd
P.1^b^	2/2	1/1	nd	nd

*ND* not determined. ^a^Pooled inactivated clinical samples. ^b^Individual non-inactivated clinical samples. RNA was purified from 140 µl aliquots by using QIAamp Viral RNA kit (Qiagen) or MagMax Viral RNA Isolation Kit (Applied Biosystems) and KingFisher Apex System (ThermoFisher), and were reversed transcribed into cDNA and amplified in a one-step RT-PCR multiplex reaction with qScript XLT one-Step RT-qPCR ToughMix (QuantaBio) using nCoV N2 combined primer/probe set (Integrated DNA Technologies, IDT), and the LightCycler 480 II instrument (Roche).

As part of sample acquisition for variant testing through the NIH Variant Task Force, we obtained sequence verified remnant samples of P.1, B.1.351, B.1.617.2 from which we created pools of VOC. We then serially diluted these pools and tested alongside B.1.2 and B.1.1.7 pools that were also serially diluted. All dilutions were done using saline matrix. Results in Table 3, show that all VOC were detected in Ct ranging from 27 to 22 (Table 3).

**Table 3:** VOC: results of Abbott BinaxNOW SARS-CoV-2 point-of-care antigen test against SARS-CoV-2 variants of concern (VOC), tested at the same time.

Pango lineage	Abbott BinaxNOW results				N2 Ct (Avg)
	Result 1	Result 2	Result 3	Final Result	
B.1.2	Positive	Positive	Positive	Positive	17.03
	Positive	Positive	Positive	Positive	20.55
	Negative	Negative	Negative	Negative	24.06
B.1.1.7	Positive	Positive	Positive	Positive	20.36
	Positive	Positive	Positive	Positive	23.66
	Negative	Negative	Negative	Negative	27.36
B.1.351	Positive	Positive	Positive	Positive	20.59
	Positive	Positive	Positive	Positive	24.14
	Negative	Negative	Negative	Negative	27.66
P.1	Positive	Positive	Positive	Positive	22.80
	Negative	Positive	Positive	Positive	27.01
	Negative	Negative	Negative	Negative	30.78
B.1.617.2	Positive	Positive	Positive	Positive	19.60
	Positive	Positive	Positive	Positive	22.09
	Negative	Negative	Negative	Negative	25.60

### Lateral flow assay comparison

**Figure 1:**
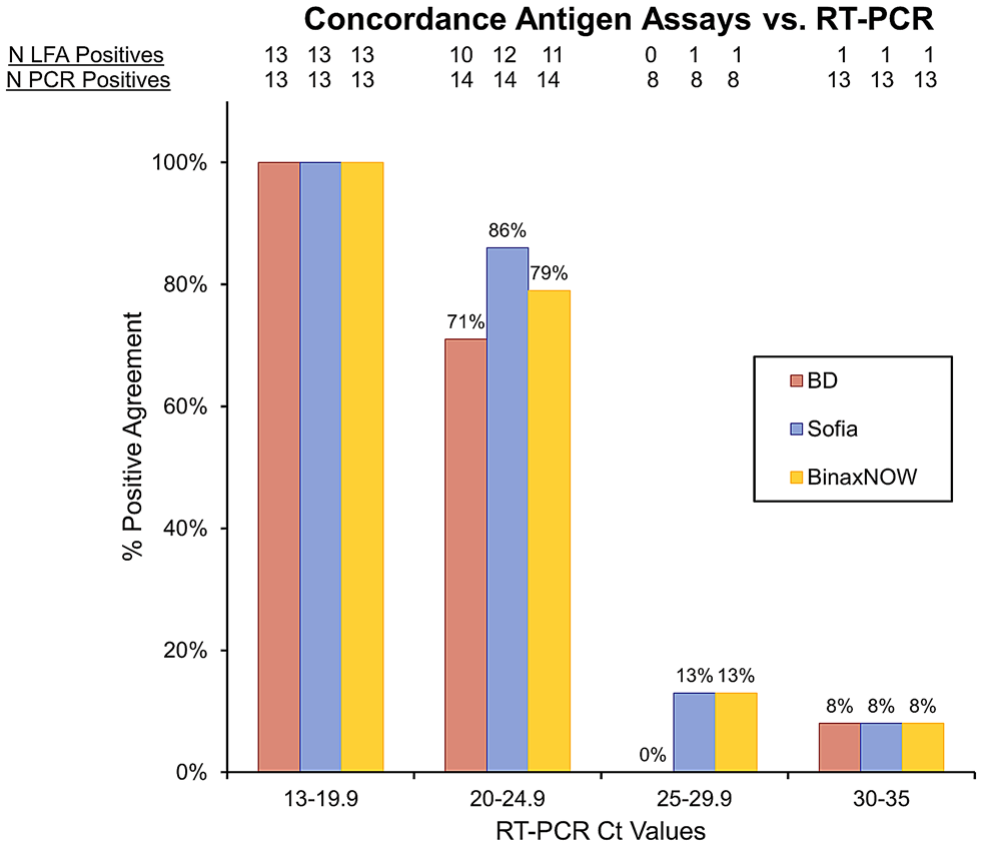
Concordance of LFA and RT-PCR results. Qualitative results from the 3 LFA assays: BD, Sofia and BinaxNOW were compared to the RT-PCR results based on the Ct values. Ct values were divided in 4 groups and displayed on the x-axis. The total number of samples tested as positive by each LFA assay is displayed at the top of the graph, with the total number of samples tested underneath. Percent (%) positive agreement of each LFA assay to the RT-PCR assay is represented on the y-axis.

Three LFA devices were each compared to the gold standard RT-PCR test and reported as percent positive agreement. We tested 48 samples from NP swabs in either saline or viral transport media. The BinaxNOW (Abbott BinaxNOW [Scarborough,MN] COVID-19 Card), Quidel Sofia2 (Quidel [SanDiego,CA] Sofia 2 SARS Ag FIA), and BD Veritor (Becton Dickinson [Sparks,MD] Veritor SARS CoV2) result concordance with RT-PCR (DiaSorin [Cypress,CA], Simplexa COVID-19 Direct) was 54%, 56%, and 50%, respectively. All three devices were 100% concordant with RT-PCR results for samples with Ct values < 20. The BinaxNOW was 79% concordant for Ct values between 20–24.9, 13% concordant between 25 and 29.9 and 8% concordant for Ct values between 30 and 35 (Fig. 1).

### Clinical performance

**Figure 2:**
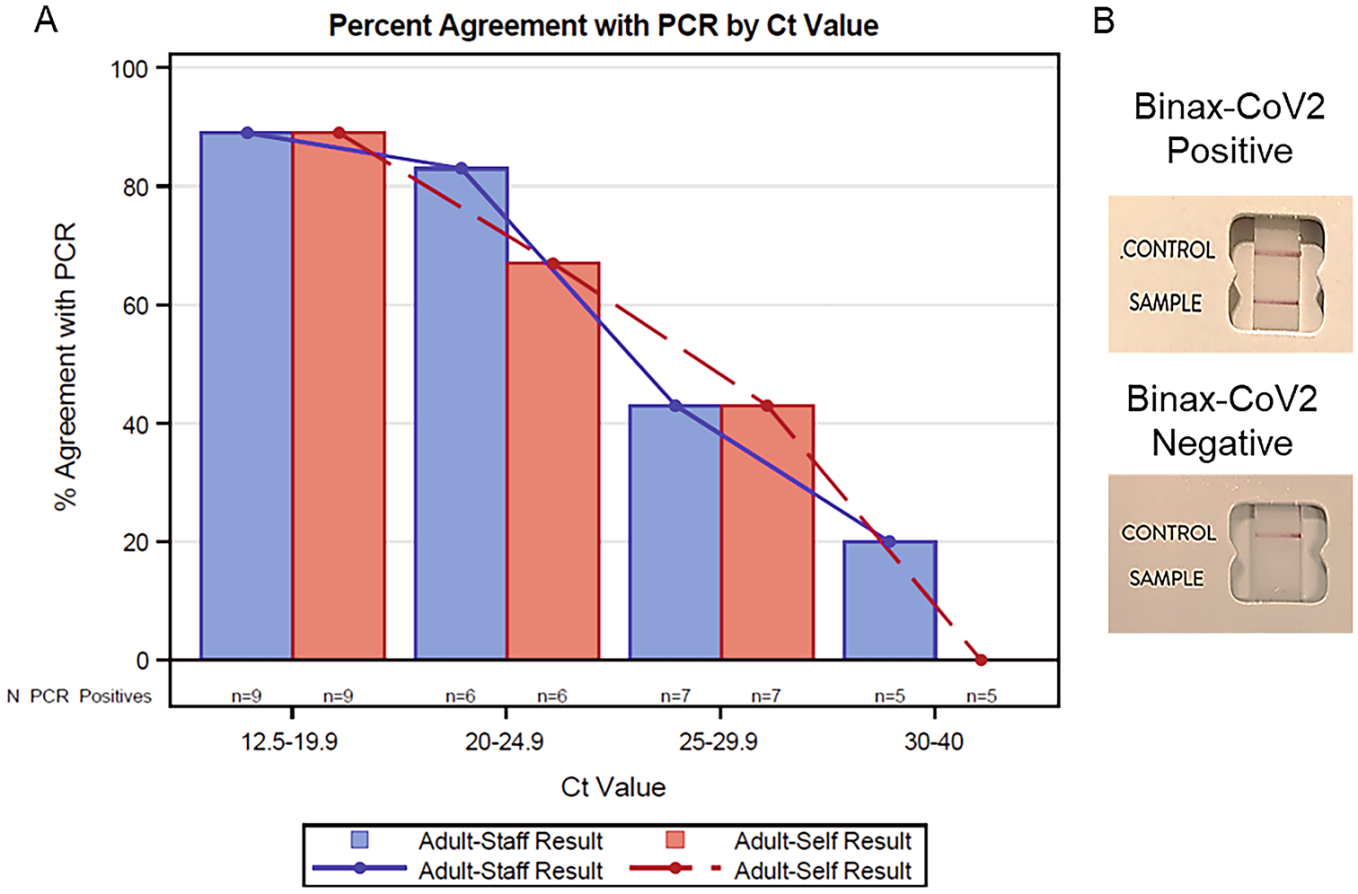
BinaxNOW agreement versus gold standard PCR decreases with rising Ct values and self collected samples. (**A**) Collection of diagnostic samples from the anterior nares was completed by either trained healthcare workers (blue) or individual participants (red). Results of quantitative RT-PCR are presented as Ct scores and used to stratify the presented data. % Agreement was calculated as the percentage of concordant test results per group. (**B**) Pictures of BinaxNOW results for positive and negative findings.

There were 309 participants included for this analysis. Cohort demographics, including participant age, sex, race, and number of days since symptom onset are included in Supplemental Table S1. For the 297 staff-collected anterior nasal (AN) swabs (77 positive), the BinaxNOW test sensitivity was 74% (95% CI 64–82%) and specificity was 99% (97–100%) compared to standard-of-care RT-PCR. The sensitivity for self or parent collected AN swabs (n = 44, 16 positive) was lower at 57% (37–76%) and specificity was 100% (79–100%). The difference between staff-collected test sensitivity and self- or caregiver-collected test sensitivity was not statistically significant (p = 0.10). When separated by age, the staff collected adult test sensitivity was 67% (52–80%) and the pediatric was slightly higher at 80% (66–89%) (p = 0.18) and specificity was 98% (87–100%) and 100% (98–100%), respectively. The kappa coefficient (95% CI) for the agreement between the adult staff collected sample versus the adult self-collected sample (n = 40) was 0.85 (0.68–1.00). The percent agreement between adult staff collected and self-collected across Ct value groups are shown in Fig. 2. There was a significant trend in decreasing agreement across increasing Ct groups (p < 0.01). We also performed sensitivity analyses by both median determined symptom onset (< and ≥ 4 days and per the BinaxNOW package directions which indicated within 7 days of symptom onset. (Supplemental Tables S2–S5).

A longitudinal case series (n = 1) found equivalent performance of the BinaxNOW and Accula PCR tests (Supplemental Figure S1).

### Test usability

Test usability was evaluated in both parents/caregivers of pediatric participants and adult participants that self-collected. Parents/caregivers of 20 participants consecutively presenting to the Children’s Healthcare of Atlanta drive-through testing site were evaluated. Three participants, aged 15–18 years, self-collected the sample and performed the test on themselves. Participants acting as caregivers collected the sample from their children (n = 17), aged 22 months–14 years, and then performed the test. Adult participants (n = 42) at Grady Hospital (22 males; mean = 56.33, SD = 16.11; range 25.36–88.9) self-collected their sample, with healthcare practitioners subsequently conducting the second antigen test for comparison in collection type.

Overall, observed parent/caregiver participants administering BinaxNOW found the test to be easy to conduct. Users noted difficulty understanding the BinaxNOW instructions, with many taking long pauses between steps to re-read. Errors were most frequently observed while users squeezed drops into the device and while inserting the swab into the device. Some users struggled with how to insert the swab, frequently checking the instructions again, with some still inserting the swab incorrectly. Some users found the dropper difficult to squeeze. Applying an uncomfortable amount of force to the dropper caused some users to miss the well and drop the buffer onto the card. For participants that tested more than one child, the second test went more smoothly and with fewer errors than the first.

**Figure 3:**
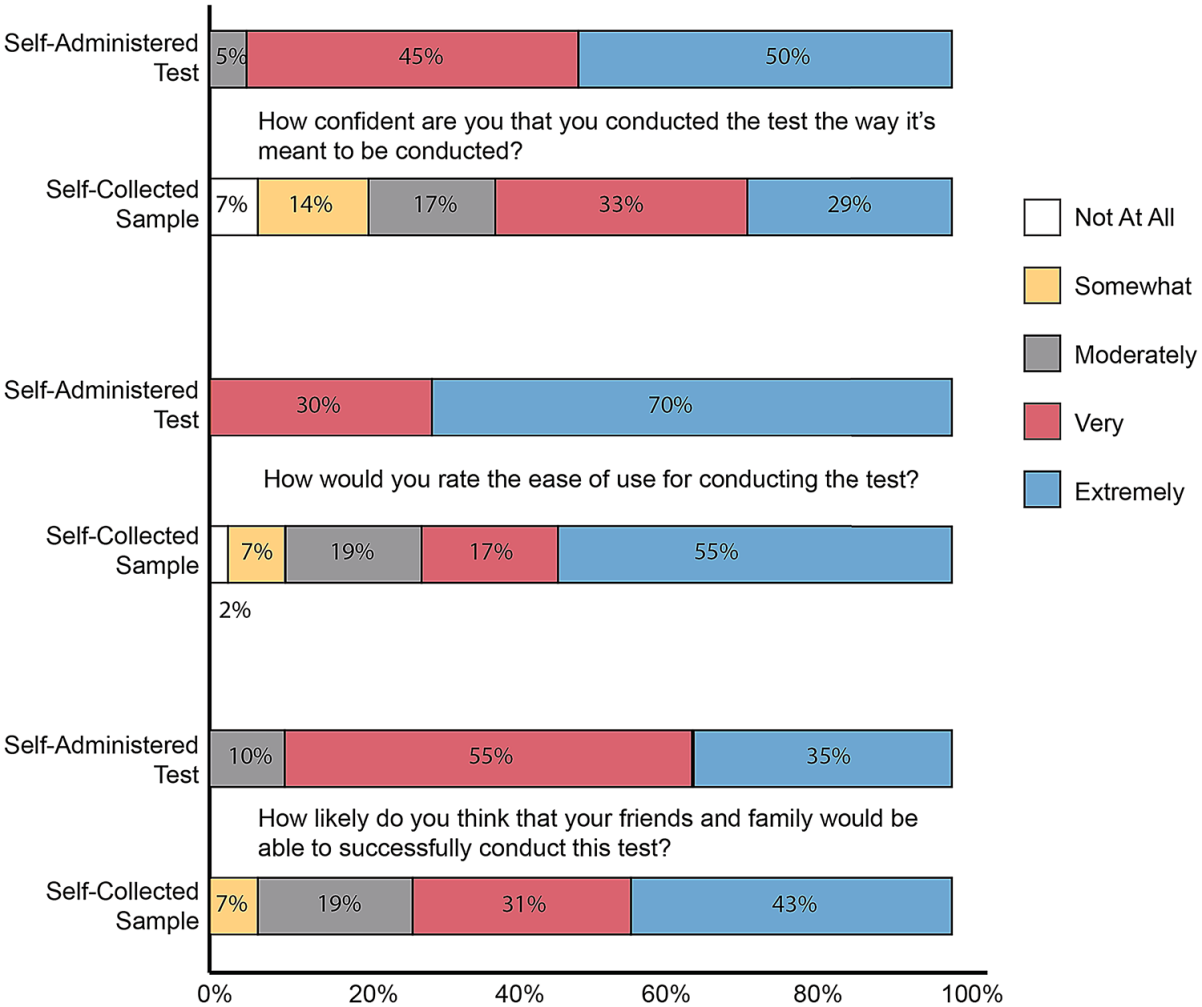
Evaluation for the independent use of BinaxNOW. Usability assessment following self-administration, with parent/caregiver (n = 17) or oneself (adolescent) (n = 3) and adult self-collected sample (n = 42). Self-collection reflects confidence in sample collection. Self-administration reflects confidence in completing assay (assay development, interpretation, etc.).

All independent users (parents/caregivers) rated BinaxNOW very or extremely easy to use, with 50% reporting that the test was extremely easy to use (Fig. 3; self-administered test data). Users were at least moderately confident that they conducted the test as intended, and 70% were extremely confident. When asked the likelihood that a user’s friends and family would be to successfully conduct this test (a question that often uncovers previously unvoiced doubts), all users reported this was moderately likely or higher, though only 35% of users stated this was extremely likely.

For users who self-collected their sample with verbal instructions from a practitioner (adult participants), scale results (Fig. 3; self-collected sample data) skewed lower. When asked about the ease of use and confidence during use, some users reported “not at all” or “somewhat” responses, with only 29% extremely confident and 55% who found the sample extremely easy to collect. Users had more confidence in their friends and families, however, as all users reported it was at least somewhat likely their peers could successfully collect the sample.

## Discussion

BinaxNOW is an emerging assay that recently received EUA for at-home, over-the-counter diagnosis of COVID-19. Clinical performance of BinaxNOW appears to be dependent on viral load. While an overall sensitivity of 74% (95% CI 64–82%) was observed following healthcare worker collection, this varied from 100% with Ct values < 14, to < 20% with Ct values > 30 (Fig. 2). Diminished test sensitivity associated with high Ct values likely reflects viral levels below the BinaxNOW LOD, which was determined to be between 94 and 750 TCID_50_/swab (2.98 × 10^3^–7.12 × 10^4^ copies/swab) with 4 different isolates (Table 1). Similar trends of decreasing sensitivity with increasing Ct values were found between the BinaxNOW, BD, and Sofia LFA assays, the latter two of which require specialized equipment for test performance (Fig. 1). No differences were found between the longitudinal home-based performance of BinaxNOW and Accula PCR based-assay when used by a single patient.

Accessibility to a variety of testing options, from rapid at-home tests to molecular PCR, is critical for COVID-19 surveillance. While sensitivity between these testing options differ, access to convenient testing options for a variety of use cases will promote maintenance testing going forward. Americans are willing to seek testing when feeling ill, with 68% preferring a home test kit to a clinic or drive through^12^. This evidence disagrees with previous studies involving HIV at home testing kits once those were widely available. Greensides et al. found that among those at high risk for HIV infection, at home and rapid HIV tests were underutilized, most citing mistrust in the results^13^. Other investigations found similar results also citing cost and lack of counseling. There is higher acceptability and willingness to use a multitude of specimen types for COVID-19 at-home testing (nasal swab, saliva)^12, 14^.

Herein, we report for the first time, the ability of Binax-CoV-2 Ag test to detect the variant of concern B.1.1.7. This variant is highly transmissible, being first identified in the UK on September 20, 2020, and a few months later became the dominant strain in the country. It contains ‘signature’ mutations in the SARS-CoV-2 spike protein, including the 69–70 deletion and two non-synonymous mutations at positions 203 and 204 of the nucleocapsid gene. Only after extensive sequencing analysis of samples with RT-qPCR negative results for S gene but positive for other genes, it has been shown that this variant was first introduced in the U.S. late November 2020, and since then it has rapidly spread out to > 30 states as of January^15^. Most importantly, a large study in the UK with clinical samples demonstrated that there is an increased risk of mortality in patients infected with B.1.1.7 versus other previously identified variants^16^. In the present study, we compared the results of BinaxNOW for B.1.1.7 and B.1.2 antigens detections, and the latter has become prevalent in the U.S and we named it as non-VOC comparator. BinaxNOW detected both lineages similarly and the Ct values of the positive analytes were ≤ 27, indicating a good performance of this rapid antigen test for its accuracy in detecting both B.1.1.7 as well as B.1.2. Moreover, BinaxNOW also detected other three major VOCs including the P.1, B.1.351, and B.1.617.2, at Ct values of 27, 24, and 22, respectively.

This study expands the external evaluation of BinaxNOW sensitivity beyond what has been previously reported. While several prior studies define test sensitivity in various real-world populations, these have omitted a usability assessment for self/caregiver collection, as well as evaluation of patients < 10 years old^10, 11, 17^. Pilarowski et al. utilized a community-based testing platform in San Francisco, CA, to report a test sensitivity of 95.4% (95% CI 90.2–98.3) among patients meeting current criteria for use of BinaxNOW (onset of symptoms associated with COVID-19 ≤ 7 days)^10^. Test sensitivity increased to 100% (95% CI 97.0–100.0) when excluding samples with an associated Ct score > 35, consistent with our findings of decreasing test sensitivity with decreasing viral loads. These findings are further supported by our LOD analysis, which suggests that lower levels of viral burden (associated with Ct scores > 35) are beyond the LOD for this particular test. Importantly, we found similar decreases in test sensitivity among all evaluated, commercially available LFAs. BinaxNOW not only performs as well as other LFAs on the market, but it is also less expensive, does not require a large electronic reader and already has an at-home EUA. Although not evaluated in this study, the CDC MMWR report on the BinaxNOW reported a low sensitivity in asymptomatic participants (35.8%) which it now has an EUA approval^11^. Sensitivity in symptomatic participants, collected by a trained researcher was 64.2% compared to our 74% in a larger sample^11^. The culmination of these findings further supports the use of antigen-based LFAs as screening tools rather than definitive diagnostic tests for the presence of SARS-CoV-2.

This study is the first to report the impact of self-collection on assay performance by both individual patients and non-medically trained caregivers. Even with in-person, guided instruction on sample collection, there was a trend toward decreased BinaxNOW sensitivity when performed by an individual patient/caregiver versus a trained healthcare worker (57% vs. 74%, p = 0.10). Although this did not reach traditional statistical significance in the current study, this drop in sensitivity would be relevant for clinical management and outpatient testing strategies if confirmed in a larger cohort. Arguments for expanded use of rapid diagnostics for SARS-CoV-2 have been based on higher sensitivity estimates from testing performed by trained medical staff^18–20^. A recent modeling study of SARS-CoV-2 testing strategies estimated test sensitivity > 95% between days 5 and 12 post infection^19^, and an earlier assessment used 70% sensitivity in models of a “poorly sensitive test”^20^. However, our data demonstrate the need for rigorous monitoring of test performance as devices are rolled out for patient/caregiver use. This may be particularly true for assays such as the BinaxNOW where the quality of sample collection is not evaluated by a specific control.

Structured usability assessments, that measures the ease of use regarding the instructions and use of the device, were supportive of patient/caregiver use (Fig. 3). This evaluation, as shown in the supplemental text, provides a rigorous template for the completion of home use assessment for emerging point-of-care testing devices. Despite that the study findings support a simple workflow and the untrained user feels confident in conducting the test alone, the reduction in sensitivity is striking. As home use of these rapid COVID-19 tests will involve completely untrained users, structured appraisal of usability and patient/caregiver feedback during sample self-collection and assay steps should be included as part of the comprehensive testing for all tests being designed for point-of-care and home use claims.

In addition, our data have clear clinical and public health implications regarding the use of these rapid COVID-19 tests like the BinaxNOW. The sensitivity of these types of tests are known to be lower than RT-qPCR, which the medical community is willing to tolerate because these devices are so much cheaper, more available, and easier to use than molecular tests, especially if they are used multiple times over several days in the same patient. Our results, however, indicate that home use of these rapid COVID-19 diagnostics as self/caregiver-operated tests may decrease this already lowered sensitivity even further. As such, this is an issue that must be considered as society collectively increases the use of these types of tests to return to “normal.”

There are several limitations to the current study. Test evaluations were performed at ambulatory testing sites and inpatient hospitals, which differs from the environment for a home use scenario. Finally, this study was designed to evaluate BinaxNOW performance among subjects currently meeting criteria for use of this assay, thereby eliminating asymptomatic carriers from this study.

This real-world, expansive age range cohort evaluation of the BinaxNOW test found significantly decreased test sensitivity with decreasing viral load (p < 0.01) and consistent performance with emerging variants. The coupling of rapid, cheap and simplicity may not be the best option for widespread community testing. The clinical significance of further decreasing diagnostic sensitivity when in the hands of an untrained user causes concern. Health providers should use caution when interpreting results from these home-use, rapid LFA tests and seek more confirmatory testing in questionable situations. This study further highlights the need for structured evaluation of home use scenarios to comprehensively evaluate future point-of-care and home use testing options that includes comparison to RT-qPCR. This assessment addresses a critical need as regulatory agencies look to expand testing access by granting approvals for use of diagnostic assays in untrained settings. It is our hope that the methodology employed in this study may be replicated to comprehensively evaluate future diagnostic assays.

## Methods

The study protocol was approved by the Emory Institutional Review Board, Children’s Healthcare of Atlanta and the Grady Research Oversight Committee. All methods were carried out in accordance with current guidelines and regulations. Detail methods used in this comprehensive evaluation are located in the supplementary materials.

BinaxNOW LOD was determined by using serial dilutions of live SARS-CoV-2 stocks of known concentrations (TCID_50_/mL and RNA copies/mL). Virus stock aliquots were thawed and serially diluted in human pooled negative nasal matrix diluted in saline (NM). Twenty microliters of each dilution were applied on the swab provided in the BinaxNOW kit. Test development was completed following instructions in the BinaxNOW package insert^8^. The results were visually interpreted according to the test lines. All tests with live SARS-CoV-2 were conducted in a BSL-3 facility.

Variant of concern (VOC) testing was assessed using remnant saline samples of NP/mid-turbinate swabs known positive for B.1.1.7, B.1.351, P.1, B.1.617.2, and B.1.2 (a non-VOC substrain of B.1) by sequencing. Each pool was prepared by combining 7–10 heat-inactivated remnant clinical samples in saline, whose sequences were determined by deep sequencing at Helix Labs (San Diego, CA). Serial dilutions of each pool were made in sterile Remel saline. Each sample was tested in triplicate. For each test, 20 μl of sample was added to swab, and IFU followed. After the 15-min incubation, results were recorded. All tests were valid, as indicated by the presence of the control line. Tests were recorded as valid and positive when a control and test line were visible. RNA was purified from each dilution and used to verify the RT-PCR Ct value using the nucleocapsid (N2) CDC primers/probe set (Table 2).

For antigen assays, manufacturer supplied swabs were immersed in clinical specimens of known Ct (DiaSorin, Simplexa) values. We tested 48 samples from NP swabs in either saline or viral transport media collected between July and August 2020. While the designated specimen for each kit is a nasal swab that is processed directly in the assay, we mimicked specimen procurement with a NP swab sampled in a clinical specimen of known viral burden to assess the sensitivity of the assay. All samples were from a single thaw after being frozen at − 80 °C. Swabs from each kit were immersed in the transport media and swirled 10 times before testing. PCR was performed to assess viral burden (assessed by CT value) and performance of the antigen detection assays. The assays were performed as specified by the product inserts. The LFA comparison data were analyzed by comparing the positive results from the three antigen tests listed in the results to RT-PCR results for the same samples and divided them by the Ct values where the positive result came up in the RT-PCR test (Fig. 1).

Clinical samples (n = 309) for test performance were collected from RADx testing sites between November 2020 and January 2021 using the printed BinaxNOW inclusion and exclusion criteria and instructions in participants aged 7 years and older that had been symptomatic fewer than 7 days. All participants and parents/guardians provided informed consent. Caregivers of children or adult participants were evaluated for self-administration data. An RT-PCR nasopharyngeal SARS-CoV-2 standard of care test was administered within 24 h of study enrollment. FDA approved RT-PCR assays included the Cobas 6800 (Roche Diagnostics, Rotkreuz, Switzerland), Abbott Alinity (Abbott Labs, Abbott Park, IL) and the Panther Fusion (Hologic, Marlborough, MA).

Usability researchers evaluated different user groups’ experiences with the BinaxNOW system, including self-sample collection, fully self-administered tests, and parent/caregiver users; these evaluations consisted of observations and Likert-type scale questions. For the statistical analysis, sensitivity and specificity estimates were reported and compared (BinaxNOW antigen result vs RT-PCR result; adult vs pediatric BinaxNOW antigen results) using Fisher’s exact tests. Percent positive agreement of antigen results vs. RT-PCR result was calculated overall, as well as Cohen’s Kappa coefficients for assessing agreement of staff vs. self-collected adult specimens.

## Supplementary Information

Supplementary Information 1.
