# Supplementary material for: Multidisciplinary assessment of the Abbott BinaxNOW SARS-CoV-2 point-of-care antigen test in the context of emerging viral variants and self-administration
Source: Sci Rep. 2021 Jul 16;11:14604. doi: 10.1038/s41598-021-94055-1 (PMC8285474; doi:10.1038/s41598-021-94055-1)
Supplement: Supplementary file 1 — Supplementary Information 1. [file 41598_2021_94055_MOESM1_ESM.pdf]

Multidisciplinary Assessment of the Abbott BinaxNOW SARS-CoV-2 Point-of-Care Antigen Test in the Context of Emerging Viral Variants and Self-Administration

Jennifer K. Frediani, PhD*[1,2], Joshua M. Levy, MD*[2,3], Anuradha Rao, PhD[2,4], Leda Bassit, PhD[2,5], Janet Figueroa, MPH[2,4], Miriam B. Vos, MD[2,4,6], Anna Wood, MPH[2,4], Robert Jerris, PhD[2,6,7], Van Leung-Pineda, PhD[2,6,7], Mark D. Gonzalez, PhD[2,6,7], Beverly B. Rogers, MD[2,6,7], Maud Mavigner, PhD[2,4], Raymond F. Schinazi, PhD[2,4], Nils Schoof, BS[2,4], Jesse J. Waggoner, MD[2,8,9], Russell R. Kempker, MD[2,8], Paulina A. Rebolledo, MD[2,8,9], Jared W. O'Neal, MPH[2,8], Cheryl Stone, RN[2,6], Ann Chahroudi, MD, PhD[2,4,6], Claudia R. Morris, MD[2,4,6], Allie Suessmith, BS[2,4], Julie Sullivan, BA[2,4], Sarah Farmer, BS[2,10], Amanda Foster, MS[2,10], John D. Roback, MD, PhD[2,7], Thanuja Ramachandra, MBBS, DCP[2,8], CaDeidre Washington, AS[2,8], Kristie Le, BS[2], Maria C. Cordero, BS[2,4], Annette Esper, MD[2,8], Eric J Nehl, PhD[2,9], Yun F Wang, PhD[2,7], Erika A. Tyburski, BS[2,10], Greg S. Martin, MD**[2,8], Wilbur A. Lam, MD, PhD**[2,7,11,12]
*shared first authors

[1] Nell Hodgson Woodruff School of Nursing, Emory University, [2]The Atlanta Center for Microsystems-Engineered Point-of-Care Technologies, [3] Department of Otolaryngology-Head & Neck Surgery, Emory University School of Medicine, [4]Department of Pediatrics, Emory University School of Medicine, [5]Laboratory of Biochemical Pharmacology, Emory University, [6]Children's Healthcare of Atlanta, [7]Department of Pathology and Laboratory Medicine, Emory University School of Medicine, [8]Department of Medicine, Emory University School of Medicine, [9]Rollins School of Public Health, Emory University [10]Georgia Institute of Technology, [11]Aflac Cancer & Blood Disorders Center at Children's Healthcare of Atlanta, [12]Wallace H. Coulter Department of Biomedical Engineering, Emory University and Georgia Institute of Technology

**Co-Corresponding authors:
Wilbur A. Lam
Aflac Cancer & Blood Disorders Center at Children's Healthcare of Atlanta
Emory University School of Medicine
Wallace H. Coulter Department of Biomedical Engineering, Georgia Institute of Technology
The Atlanta Center for Microsystems-Engineered Point-of-Care Technologies
Office: (404) 727-7473
Wilbur.lam@emory.edu

Greg S. Martin
Department of Medicine
Emory University School of Medicine
The Atlanta Center for Microsystems-Engineered Point-of-Care Technologies
Greg.martin@emory.edu

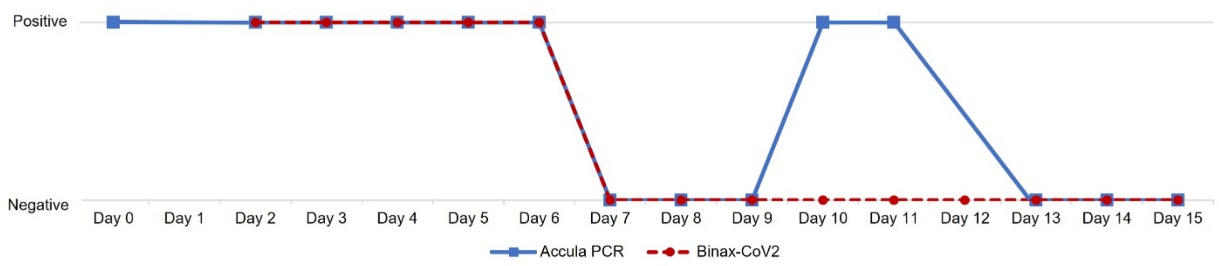

Supplemental Figure 1. Longitudinal Comparison (n=1) of Binax-CoV2 versus Diagnostic PCR. Longitudinal comparison of the Accula PCR (Mesa Biotech) to the Binax-CoV2. One asymptomatic participant performed both the point-of-care Accula PCR (blue) and Binax-CoV2 antigen test (red) for 15 days. Results were comparable and usability was similar to adult participants who self-collected.

Supplemental Table 1. Test Performance by BinaxNOW Directed Symptom Onset Days – Staff-Collected

	**Sensitivity (Exact 95% CI)**	**Specificity (Exact 95% CI)**	**Kappa (Exact 95% CI)**
**Overall (N=297)**	74% (64%-82%)	99% (97%-100%)	0.78 (0.70-0.86)
**<7 symptom onset days (N=270)**	77% (67%-85%)	99% (97%-100%)	0.81 (0.73-0.88)
**7 symptom onset days* (N=27)**	50% (21%-79%)	100% (78%-100%)	0.53 (0.23-0.82)

*Study was limited to ≤7 symptom onset days

Supplemental Table 2. Test Performance by BinaxNOW Directed Symptom Onset Days – Self-Collected

	**Sensitivity (Exact 95% CI)**	**Specificity (Exact 95% CI)**	**Kappa (Exact 95% CI)**
**Overall (N=44)**	57% (37%-76%)	100% (79%-100%)	0.49 (0.28-0.70)

**<7 symptom onset days (N=38)**	56% (35%-76%)	100% (75%-100%)	0.47 (0.24-0.69)
**7 symptom onset* days (N=6)**	67% (9%-99%)	100% (29%-100%)	0.67 (0.10-1.00)

*Study was limited to ≤7 symptom onset days

Supplemental Table 3. Test Performance by Median Symptom Onset Days – Staff-Collected

	Sensitivity (Exact 95% CI)	Specificity (Exact 95% CI)	Kappa (Exact 95% CI)
**Overall (N=297)**	74% (64%-82%)	99% (97%-100%)	0.78 (0.70-0.86)
**<4 symptom onset days (N=168)**	76% (63%-87%)	100% (97%-100%)	0.81 (0.72-0.91)
**≥4 symptom onset days (N=129)**	71% (56%-83%)	99% (93%-100%)	0.74 (0.61-0.86)

Supplemental Table4. Test Performance by Median Symptom Onset Days – Self-Collected

	Sensitivity (Exact 95% CI)	Specificity (Exact 95% CI)	Kappa (Exact 95% CI)
**Overall (N=44)**	57% (37%-76%)	100% (79%-100%)	0.49 (0.28-0.70)

**<4 symptom onset days (N=19)**	54% (25%-81%)	100% (54%-100%)	0.42 (0.11-0.74)
**≥4 symptom onset days (N=25)**	60% (32%-84%)	100% (69%-100%)	0.55 (0.26-0.83)

Methods:

Cells and Virus

Vero E6 cells (CRL-1586, ATCC, Manassas, VA, USA) were cultured in Dulbecco's Modified Eagle Medium (Corning, Corning, NY, USA) supplemented with 10% heat inactivated fetal bovine serum (Gemini Bio-Products, Sacramento, CA, USA) and 1% penicillin/streptomycin (Corning, Corning, NY, USA). Cells were incubated at 37°C in 5% CO_2. SARS-CoV-2 isolates USA-WA1/2020, USA-CA3/2020 and Italy-INMI1 were obtained from BEI Resources. USA-GA4/2020 was isolated from a PCR-positive nasopharyngeal swab collected at Emory University from a patient hospitalized with COVID-19 in July 2020. The swab was placed in virus transportation medium immediately after collection, the virus-containing medium was isolated by centrifugation and aliquots were stored at -80°C until further use.

Virus stock preparation

Vero E6 cells at 90% confluence were infected at a multiplicity of infection (MOI) of 0.001-0.1 with the various isolates. After 1h at 37 °C, fresh medium was added to the cell culture and cytopathic effect (CPE) were monitored every 24h until reaching > 50% CPE. Culture supernatants were collected and clarified through centrifugation and aliquots were stored at -80°C. Second passage viral supernatants were titrated by Median Tissue Culture Infectious Dose ($TCID_{50}$) assay and by real-time qPCR targeting the nucleocapsid N2 gene and sequenced via direct deep sequencing on an Illumina MiSeq (Illumina, Inc., San Diego, CA).

These stocks were used for device testing.

LOD evaluation with live virus

BinaxNOW LOD was determined by using serial dilutions of live SARS-CoV-2 stocks of known concentrations ($TCID_{50}$/mL and RNA copies/mL). Virus stock aliquots were thawed and serially diluted in human pooled negative nasal matrix diluted in saline (NM). Twenty microliters of each dilution were applied on the swab provided in the BinaxNOW kit. Test development was completed following instructions in the BinaxNOW package insert.[1] The results were visually interpreted according to the test lines. All tests with live SARS-CoV-2 were conducted in a BSL-3 facility.

Variant Testing

The Variant Task Force (VTF), working with Helix (San Diego, CA) obtained remnant clinical samples (NP/mid- turbinate swabs in saline) that are confirmed positive for SARS-CoV-2 VOC by RT-PCR and whole genome sequencing. These samples are inactivated at Helix by heating to 75^0C for 30 minutes. The goal is to build a sample bank at ACME, containing VOC and non-VOC comparators that are ready to use for evaluating diagnostic tests. For data obtained using pooled samples in Table 2, we pooled six samples of low Ct values (≤ 16) each of B.1.1.7 (VOC) and B1.2 (a non-VOC comparator). These pools were serially diluted in negative matrix containing 15,000 human adenocarcinoma alveolar basal epithelial A549 cells (ATCC) per ml phosphate buffer saline (PBS), and an aliquot of each was assessed by viral RNA isolation and RT-PCR for CDC-N2 gene. Each diluted sample was evaluated using the BinaxNOW COVID-19 Ag Card for ability to detect SARS-CoV-2 Ag. In addition, non-inactivated undiluted remnant clinical samples tested positive (RT-PCR and sequencing) for B.1.2, B.1.1.7, B.1.351 and P.1 were evaluated with BinaxNOW.   For data shown in Table 3, pools were created using 8-10 heat inactivated patient samples. These were serially diluted in saline matrix.

Lateral Flow Assay Comparison

To give insight into the comparative performance between several commercially available SARS-CoV-2 antigen-based lateral flow assays (LFA), the cycle threshold (Ct) values of a PCR-based molecular test (DiaSorin [Cypress,CA], Simplexa COVID-19 Direct) were compared to three LFAs with current FDA EUAs (Abbott BinaxNOW [Scarborough,MN] COVID-19 Card, Becton Dickinson [Sparks,MD] Veritor SARS CoV2, and Quidel [SanDiego,CA] Sofia 2 SARS Ag FIA) to determine a cutoff value for LFA positivity.

Samples were selected by a blinded study coordinator from Children's Healthcare of Atlanta clinical samples. Patients had a wide range of symptoms from none to case definition COVID-19 symptoms. All assays were performed on the same day within a 3-hour period. Ct values for S and Orf1ab genes were averaged for a single comparative value. Discrepant results were repeated.

Human Subject Research

The Atlanta Center for Microsystems Engineered Point-of-Care Technologies (ACME-POCT) network utilized hospital and community-based COVID-19 testing centers for enrolling patients. Point-of-care testing sites included Emory University Hospital, Emory University Hospital Midtown, Grady Memorial Hospital, and the Children's Healthcare of Atlanta drive-through testing center. Eligible participants were identified consecutively at each study site via review of COVID-19 symptoms. An RT-PCR nasopharyngeal SARS-CoV-2 standard of care test was administered within 24 hours of study enrollment. FDA approved RT-PCR assays included the Cobas 6800 (Roche Diagnostics, Rotkreuz, Switzerland), Abbott Alinity (Abbott Labs, Abbott Park, IL) and the Panther Fusion (Hologic, Marlborough, MA). Exclusion criteria included patients who were asymptomatic, had symptoms associated with COVID-19 for $\geq$ 7 days, were unable to tolerate an anterior nares swab, or were unable to provide informed consent.

Symptom criteria were chosen to reflect published indications for the use of BinaxNOW.[1] Ambulatory testing centers enrolled consecutive persons under investigation for assessment of test specificity. An additional patient was followed longitudinally with daily self-completion of BinaxNOW and Accula PCR (Mesa Biotech, San Diego, CA) testing in the home setting. Clinical and demographic variables were collected in a centralized, web-based database (REDcap, Nashville, TN).[2] The study protocol was approved by the Emory Institutional Review Board, Children's Healthcare of Atlanta and the Grady Research Oversight Committee.

Test Performance

Following identification, eligible participants were approached by study personnel, who obtained informed consent. Clinical electronic case report forms were used to collect demographic and clinical variables, including age, sex, race, COVID-19 related symptoms and time of symptom onset, current health conditions and exposure status. Bilateral anterior nasal samples were collected according to the BinaxNOW package insert.[1] The assay was subsequently completed at the point-of-care by the study coordinator, with photo documentation of the test results for quality assurance.

Usability Assessment

The Georgia Tech HomeLab initiative completed BinaxNOW usability assessment for the completion of self-collection by both individual patients, as well as non-medically trained caregivers.[3] Two usability researchers observed the test at the Children's Health Care of Atlanta drive-through testing site. While evaluating the device, the researchers considered the use case for home users. This would include users with no training on the device and who would not perform a large volume of tests in any given period, i.e., individuals testing themselves or parents testing their child. Data collection occurred via observation, semi-structured interview, and Likert-type scale questions.[1] Each participant was given the BinaxNOW Quick Reference

Guide, then asked to complete the assay protocol. Participants were guided on sample collection as there were no instructions provided for that step. After the process was complete, the participants were asked two open-ended questions and a series of Likert-type scale questions designed to evaluate user confidence and ease of use. Each question was read out loud and the participant was asked to choose a response based on a paper scale placed in front of him/her.

Additionally, patients at Grady Hospital were given verbal instructions on how to self-collect a sample for this test. Practitioners instructed patients and then administered the same series of Likert-type scale questions that participants at the CHOA drive-through responded to. Results of the comprehensive usability evaluation are provided in the Online-Only Supplement.

Document Control No: RADX-016-0-0001
Version Control No.: 1

AbbottBinaxNOW™

COVID-19 Ag Card

Usability Report

Georgia Tech HomeLab
Georgia Tech Center for Advanced Communications Policy

February 2020

Georgia Tech Center for Advanced Communications Policy

Georgia Tech HomeLab

1 Contents

1 Contents 1
2 Executive Summary 3
3 Methods 3
3.1 Lab Observation 3
3.1.1 Protocol 3
3.1.2 Limitations 3
3.2 Expert Evaluation....................4
3.2.1 Simulated Use Cases 4
3.2.2 Limitations 4
3.3 Novice Users 5
3.3.1 Protocol 5
3.3.2 Limitations 5
4 Results....................6
4.1 Lab Observation 6
4.1.1 Time on Task....................6
4.1.2 Ratings 7
4.1.3 Participant Feedback 8
4.2 Expert Evaluation....................10
4.2.1 General Feedback 10
4.2.2 Arthritis Simulation....................11
4.2.3 Low Vision Simulation....................11
4.3 Novice Users 13
4.3.1 Self-administered Tests 13
4.3.2 Self-collected Samples 14
5 Recommendations 15
6 F&F Usability Matrix....................16
6.1 Point of Care....................17
6.2 Trained Layperson....................18
6.3 Home Use....................19
7 References 21
8 Appendix A. Procedure Card....................22

Table 1: Task Duration....................6
Table 2. System Usability Scale....................8
Table 3. Arthritis Simulation Ratings....................11
Table 4. Low vision simulation results- Cataracts. 12

Table 5. Low vision simulation results- Macular degeneration 12
Table 6. Low vision simulation results- Visual acuity of 20/70....13
Table 7. Low vision simulation results- Visual acuity of 20/200....13

Figure 1: Task Duration 7
Figure 2. Scale Question Results. 8
Figure 3. Likert Scale Results for self-administered tests. 14
Figure 4. Likert Scale Results for self-collected samples. 15
Figure 6: Usability- Point of care 18
Figure 7: Usability- trained layperson 19
Figure 8: Usability- home use....20

2 Executive Summary

This usability report outlines the evaluation of the Abbott BinaxNOW™ COVID-19 Ag Card and test protocol. This evaluation consisted of:

- an expert evaluation of the system components and training materials
- an observation and interview with personnel who completed the tests in a lab setting
- an arthritis simulation to determine the usability for users with reduced strength and dexterity
- a low vision simulation to determine the usability for users with reduced visual acuity
- an observation of 20 novice users who self-administered the test
- feedback collected from 42 novice users who self-collected samples

The purpose of this usability evaluation was to determine the extent to which healthcare professionals, trained laypeople, and home users can run the Abbott BinaxNOW™ COVID-19 Ag Card protocol. The test protocol consists of the user dispensing 6 drops of reagent from a dropper bottle into the top one of two openings in the test booklet. The user will then collect a sample from the patient using a nasal swab. Once the sample is collected, the user will insert the swab into the bottom opening in the test booklet, and push it upward where the swab is seen through the top opening. While leaving the swab inserted, the user will rotate or "twirl" the swab three times to the right. Using an adhesive strip inside the booklet, the user will then secure the booklet closed. The test kit will now resemble a lollipop, as the swab will remain inside the booklet. The user will wait 15 minutes for a result to develop on the outside of the booklet. Using the instruction manual, the user will then compare possible results listed with the developed test to interpret the result.

Overall, the researchers found the device to be very usable for the healthcare setting and trained laypeople. The home setting was found to be moderately usable with the potential for increased usability if recommended changes are implemented. Although some steps in the protocol are novel to most users, researchers found users to have a short learning curve when conducting multiple tests. In all user scenarios, improved instruction materials would increase the usability greatly.

3 Methods

3.1 Lab Observation

3.1.1 Protocol

The observation occurred at the Children's Health Care of Atlanta drive-through testing site, in a temporary laboratory (housed inside a CHOA clinic onsite). The observation sessions occurred on the sixth full day of testing the device at the drive-through site. The user conducting the tests was
a laboratory director within the research unit at CHOA. A second user, a research coordinator, was also interviewed.

Two usability researchers observed the test. An evaluation protocol was developed for the specifics of this novel test and used to capture and record data. Data collection occurred via observation, semi- structured interview, the System Usability Scale, and Likert-type scale questions.

3.1.2 Limitations

This evaluation was an observation of the test procedure as the user conducted the tests and an internal evaluation of the device before the lab observation. The lab observation was limited to a trained user after multiple days of performing the test. Report data is based on the attitudes and experiences vocalized by the user, quantitative results from Likert-type scale questions, and insights noted by researchers.

3.2 Expert Evaluation

During this evaluation, a usability researcher ran through the test protocol from start to finish and documented the experience. Researchers also evaluated the training materials (Procedure Card and Product Insert). A list of pros and cons were identified, and recommendations were made where possible. Finally, the researchers determined overall usability scores for three possible use cases (point of care, trained layperson, home use) by rating the level of efficiency of the device and the possibility of errors.

3.2.1 Simulated Use Cases

Researchers utilized use case simulations to better understand how certain populations would interact with the system. Simulated use cases can be utilized to identify issues that users may face early in the testing process, before full user testing occurs (Shao, 2009). Additionally, these simulations can provide insights on how users with functional limitations will interact with the system (O'Brien et al., 2015).

3.2.1.1 Arthritis

To better evaluate the accessibility of the device, a researcher used arthritis simulating gloves to run through the protocol. Due to the rapidly increasing number of consumers with arthritis, manufactures must consider the wants, needs, and abilities of individuals with arthritis. However, manufacturers and designers of consumer products often do not understand the impact of arthritis on a consumer's ability to access and use their products and/or packaging. The Arthritis Simulation Gloves were designed to simulate the loss of functional abilities associated with moderate to severe arthritis of the hand (Mann et al., 2012)

3.2.1.2 Low Vision

To better evaluate the accessibility of the device, a researcher used low vision simulation goggles to run through the protocol. Due to the rapidly increasing number of consumers with low vision, manufactures must consider the wants, needs, and abilities of individuals with low vision. However, manufacturers and designers of consumer products often do not understand the impact of low vision. on a consumer's ability to use their products and/or packaging. The low vision goggles have different lenses designed to simulate reduced visual acuity associated with cataracts, macular degeneration, 20/70 vision, and 20/200 vision (Zimmerman Low Vision Simulation Kit).

3.2.2 Limitations

These use case simulations are used as a proxy to recreate the conditions for specific groups of users, but are not intended to replace user testing with these populations. While these simulations are used to approximate the functional limitations of certain disabilities, they are not representative of the actual experiences of individuals with disabilities. The simulations were conducted by researchers who were familiar with the test protocol, and so were not representative of novice users.

3.3 Novice Users

3.3.1 Protocol

3.3.1.1 *Self-administered Tests*

While evaluating the device, the researchers considered the use case for home users. This would include users with no training on the device and who would not perform a large volume of tests in any given period, i.e., individuals testing themselves or parents testing their child.

This evaluation occurred at the Children's Health Care of Atlanta drive-through testing site. Over two days of observation, researchers observed 20 participants. Three participants, aged 15-18 years, self- collected the sample and performed the test on themselves. 17 participants collected the sample from their children, aged 22 months- 14 years, and then performed the test.

Each participant was given the Quick Reference Guide, then asked to complete the protocol. Each participant was guided on collecting the sample as there were no instructions provided for that step. After the process was complete, the participants were asked the following questions:

1. How was that process?

2. Is there anything you disliked about it?

3. On a scale of 1 to 5, how confident are you that you conducted the test the way it's meant to be conducted? 1 is not at all confident and 5 is very confident.

4. On a scale of 1 to 5, how would you rate ease of use for conducting the test? 1 is not at all easy and 5 is extremely easy.

5. On a scale of 1 to 5, how likely do you think it is that your friends and family would be able to successfully conduct this test? 1 is not at all likely and 5 is extremely likely.

3.3.1.2 *Self-collected Samples*

Forty-two patients at Grady Hospital (22 males; M_{age}= 56.33, *SD*= 16.11; Range: 25.36-88.9) were given verbal instructions on how to self-collect a sample for this test. Practitioners instructed patients and then administered the following three questions:

1. On a scale of 1 to 5, how confident are you that you conducted the test the way it's meant to be conducted? 1 is not at all confident and 5 is very confident.

2. On a scale of 1 to 5, how would you rate ease of use for conducting the test? 1 is not at all easy and 5 is extremely easy.

3. On a scale of 1 to 5, how likely do you think it is that your friends and family would be able to successfully conduct this test? 1 is not at all likely and 5 is extremely likely.

3.3.2 Limitations

The observations were conducted at a drive-through testing site which differs from the environment for a home use scenario. Considerations include: the cold outside temperatures, wind, and the possible feeling of urgency while researchers watched. These users were also aware that they would receive official test results (via rt-PCR test conducted concurrently), and as a result stakes may have been lowered.

Researchers were not present for the self-collected samples, and only received the results of the scale questions. This study examined the ease of use for the sample collection only; users did conduct the entire test protocol.

4 Results

4.1 Lab Observation

4.1.1 Time on Task

The user conducted four tests concurrently, including preparing the sample (i.e., adding extraction reagent to the card, inserting and turning swab, closing card), test run time (i.e., the 15 minutes required for the test to develop), and interpreting the results (i.e., interpreting the results presented in the card window). All tasks combined took less than 20 minutes. Notably, one of the positive samples showed the positive line after 1 of processing, but the user waited until the full 15-minute processing time was complete for each sample before officially interpreting results. Table 1 and Figure 1 show the amount of time the participant spent on each task.

Task	Length of Time
Preparing samples	3 minutes
Processing samples	15 minutes
Interpreting results	2 minutes

Table 1: Task Duration

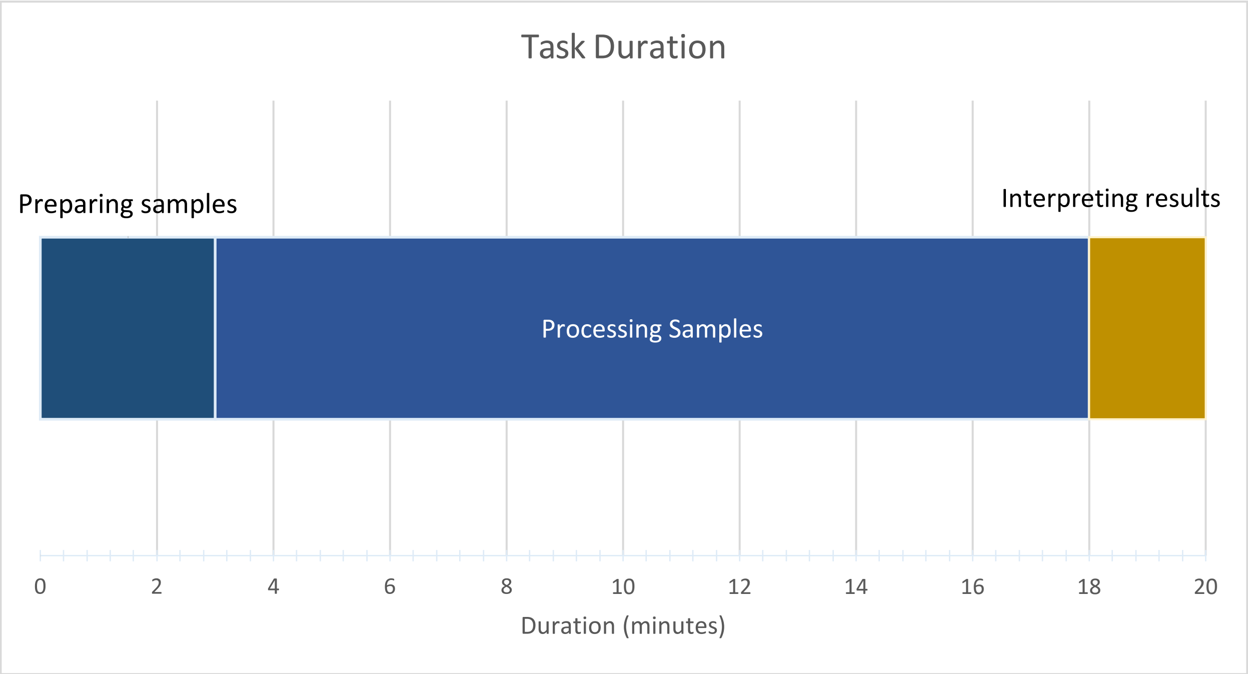

Figure 1: Task Duration

4.1.2 Ratings

4.1.2.1 System Usability Scale

The System Usability Scale (Brooke, 1986) is a quick way to subjectively capture a participant's global attitude toward a system's usability. The scale consists of ten-item Likert scale, with responses ranging from "strongly disagree" to "strongly agree." After the conclusion of the test, each item was read out loud and the participant (User 1) was asked to choose a response based on a paper scale placed in front of him/her. Items were scored and combined into one total number, which is then assigned a letter grade. Raw results are shown in Table 2. The user scored the system 95 out of 100, which is indicative of the highest possible letter grade of A (Sauro & Lewis, 2016).

	1- Strongly Disagree	2- Disagree	3- Neither Agree nor Disagree	4- Agree	5- Strongly Agree
I think that I would like to use this system frequently.					X
I found the system unnecessarily complex.	X				
I thought the system was easy to use.					X
I think that I would need the support of a technical person to be able to use this system.	X				
I found the various functions in this system were well integrated.					X

I thought there was too much inconsistency in this system.	X				
I would imagine that most people would learn to use this system very quickly.				X	
I found the system very cumbersome to use.	X				
I felt very confident using the system.					X
I needed to learn a lot of things before I could get going with this system.		X			

Table 2. System Usability Scale.

4.1.2.2 Ease of Use

After the conclusion of the test, the users were asked to respond to three Likert scale questions. The first assessed overall confidence in the user's ability to conduct the protocol according to the IFU, and two assessed the ease of use of the system. The paper response scales were placed in front of the users and they were asked to choose the response that most closely aligned with their attitudes toward the system. Responses are included in Figure 2. The users indicated that the ease of use for conducting the test was extremely easy, and interpreting test results fell between extremely and very easy. The users reported being confident that they conducted the test that way it was intended to be conducted.

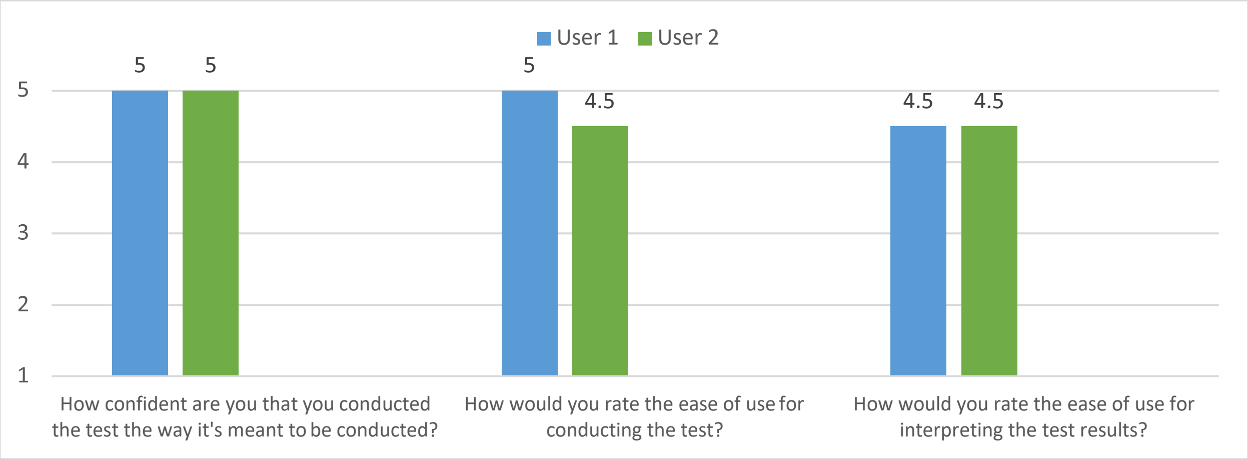

Figure 2. Scale Question Results.

4.1.3 Participant Feedback

4.1.3.1 Pros

4.1.3.1.1 Protocol

Although User 1 is trained medically, User 2 is a research coordinator without medical training. Both users found the process to be easy to complete.

"It seems pretty hard to mess it up" – User 2

4.1.3.1.2 Device

The participants appreciated the simple "popsicle" form factor.

"I really, really like the design" – User 1

One user pointed out that the reservoir for the reagent is under both holes in the card. This allows for user error in the case the user puts the drops in the bottom opening rather than the top.

The disposable nature of the device allows easy labeling- either stickers or writing directly on the card. This allows many tests to run simultaneously without risk of mixing up patient samples.

4.1.3.1.3 Results Interpretation

Overall, the users found that the results were very clear. In the case that the positive line came through slightly faint, the users noted that taking a picture of the test result more clearly showed the line.

"You know, some [tests] you have to hold up to the light and stand on one foot [to interpret the results]." "I have not questioned a single one of them. The negatives are negatives, there's no doubt about it. The positives are positive."
"No ambiguity here." – User 1

4.1.3.1.4 Instructions for Use

User 1 indicated that the instructions were useful and easy to understand.

"[The instructions were] very clear." – User 1

4.1.3.2 Cons

4.1.3.2.1 Protocol

One user noted that her first attempt at inserting the swabs into the holes was confusing and she had to reread the instructions for clarity.

"Slipping up and under was a little confusing." – User 1

Both users mentioned adding the reagent drops as being the most cumbersome step in the process. However, both mentioned this as a minor inconvenience and didn't have trouble counting the 6 individual drops. Pre-measured reagent packs or vials would enhance the user experience.

4.1.3.2.2 Device

During the observation, User 1 pointed out that she "pops" the spine of the card because it doesn't naturally lay flat when opened.

4.1.3.2.3 Results Interpretation

The users noted that in a few cases, the test strip was shifted where the control line appeared lower than it should have on the outside of the card. Users were able to manually shift the strip into the correct position.

4.2 Expert Evaluation

4.2.1 General Feedback

4.2.1.1 Pros

4.2.1.1.1 Device

The form factor is streamlined and eliminates the need for multiple pieces. This reduces possibility for error or device malfunction. Additionally, the all-in-one, “lollipop” design reduces the possibility of contamination of the environment because it is used and thrown away in one unit.

The time limits are listed for the sample eligibility and results validity. This gives the user confidence in the accuracy of the test.

4.2.1.1.2 Instructions for Use

The procedure card is detailed and anticipates possible user questions with helpful images – how far to push the swab into the hole, how many times to turn the swab, and what direction to turn the swab.
The use of bolding and numbering help the user comprehend the protocol more easily. The procedure card also clearly conveys the possible results for the user to compare during results interpretation.

4.2.1.2 Cons

4.2.1.2.1 Device

The dropper bottle requires some force to squeeze and does not have the most discrete drops. The drops sometimes pool on the dropper tip rather than creating separate drops, making it difficult to count the number of drops deposited into the device. Because of the force required to squeeze the dropper, users with reduced strength or dexterity may find this step challenging.

The hole for reagent drop is small. This will make it challenging for users with reduced visual acuity or dexterity to accurately place the drops.

Individuals with color-impaired vision may not be able to adequately interpret test results.

4.2.1.2.2 Instructions for Use

The procedure card and product insert would be especially challenging for people with reduced visual acuity, though digital materials may be accessible. The printed version needs a larger font to be more accessible to users.

The images on the procedure card are small and are not highly realistic; this could cause confusion. Larger, more realistic images would be helpful to better communicate the protocol. Additionally, the use of all caps in parts of the card reduces readability rather than create emphasis.

Because color indicates the validity of the test, individuals with color-impaired vision may not be able to adequately interpret test results.

On the procedure card, the “Important” section is too subtle and could benefit from a larger text. This could cause the user to overlook key information.

Some wording in the instructions is elevated and would benefit the user if simple, more direct language was used. For example, “Negative results, from patients with symptom onset beyond seven days, should be treated as presumptive and confirmation with a molecular assay, if necessary, for patient management, may be performed” may be hard for some users to understand.

4.2.2 Arthritis Simulation

Using the gloves, the researcher was able to complete the protocol. She was able to open the book with no difficulty. Closing the book presented little difficulty. Collecting the sample with the swab, inserting the swab into the hole, twisting the swab, and removing the adhesive liner were completed with some difficulty. Manipulating the dropper and inserting the drops into the small hole presented the user with a great amount of difficulty- causing some drops to miss the hole completely. The researcher was unable to open the swab and booklet packaging without the use of a tool. The table below presents the task and the corresponding rating for difficulty.

	No Difficulty	Little Difficulty	Some Difficulty	Great Difficulty	Unable to Complete Without Tool	Unable to Complete Task
Open Device Packaging					X	
Open Swab Packaging					X	
Collect Sample			X			
Open Device/Booklet	X					
Open Dropper Cap				X		
Squeezing Drops into Device				X		
Replacing Dropper Cap				X		
Inserting Swab into Device			X			
Twisting Swab in Device			X			
Removing Adhesive Backing			X			
Closing Device/ Booklet		X				

Table 3. Arthritis Simulation Ratings

4.2.3 Low Vision Simulation

Using the goggles with various lenses provided challenges for most tasks. Reading the Quick Reference Guide was possible to complete with some difficulty for the reduced visual acuity lenses, but was not possible for cataracts and macular degeneration lenses. Squeezing drops into the device was also challenging across the board, and impossible to complete with the macular degeneration lenses. The table below presents the task and the corresponding rating for difficulty.

	No Difficulty	Little Difficulty	Some Difficulty	Great Difficulty	Unable to Complete Without Tool	Unable to Complete Task
Read Quick Reference Guide						x
Open Device Packaging			x			
Open Swab Packaging		x				
Collect Sample	x					
Open Device/Booklet	x					
Open Dropper Cap		x				
Squeezing Drops into Device			x			
Replacing Dropper Cap		x				
Inserting Swab into Device			x			
Twisting Swab in Device	x					
Removing Adhesive Backing		x				
Closing Device/ Booklet	x					

Table 4. Low vision simulation results- Cataracts.

	No Difficulty	Little Difficulty	Some Difficulty	Great Difficulty	Unable to Complete Without Tool	Unable to Complete Task
Read Quick Reference Guide						x
Open Device Packaging			x			
Open Swab Packaging		x				
Collect Sample	x					
Open Device/Booklet	x					
Open Dropper Cap		x				
Squeezing Drops into Device						x
Replacing Dropper Cap		x				
Inserting Swab into Device						x
Twisting Swab in Device		x				
Removing Adhesive Backing			x			
Closing Device/ Booklet		x				

Table 5. Low vision simulation results- Macular degeneration.

	No Difficulty	Little Difficulty	Some Difficulty	Great Difficulty	Unable to Complete Without Tool	Unable to Complete Task
Read Quick Reference Guide			x			
Open Device Packaging		x				
Open Swab Packaging		x				
Collect Sample	x					
Open Device/Booklet	x					
Open Dropper Cap	x					
Squeezing Drops into Device		x				
Replacing Dropper Cap	x					
Inserting Swab into Device		x				
Twisting Swab in Device	x					
Removing Adhesive Backing		x				
Closing Device/ Booklet	x					

Table 6. Low vision simulation results- Visual acuity of 20/70.

	No Difficulty	Little Difficulty	Some Difficulty	Great Difficulty	Unable to Complete Without Tool	Unable to Complete Task
Read Quick Reference Guide			x			
Open Device Packaging			x			
Open Swab Packaging		x				
Collect Sample	x					
Open Device/Booklet	x					
Open Dropper Cap	x					
Squeezing Drops into Device			x			
Replacing Dropper Cap	x					
Inserting Swab into Device			x			
Twisting Swab in Device	x					
Removing Adhesive Backing		x				
Closing Device/ Booklet	x					

Table 7. Low vision simulation results- Visual acuity of 20/200.

4.3 Novice Users

4.3.1 Self-administered Tests

Overall, the users found the test to be easy to conduct, but the instructions were somewhat difficult to comprehend. Most users took long pauses between steps to read the instructions over again. Errors were most frequently observed while users squeezed drops into the device and while inserting the swab into the device. Some users struggled with how to insert the swab, frequently checking the instructions again, with some still inserting the swab incorrectly. Some users found the dropper difficult to squeeze. Applying an uncomfortable amount of force to the dropper caused some users to miss the well and drop the buffer onto the card. For participants that tested more than one child, the second test went more smoothly and with fewer errors than the first. Likert scale results are shown below (Figure 1; Self- administer data). All users found ease of use very or extremely easy to use, with 50% reporting that the test was extremely easy to use. Users were at least moderately confident that they conducted the test as intended, and 70% were extremely confident. When asked the likelihood that a user’s friends and family would be to successfully conduct this test (a question that often uncovers previously unvoiced doubts), all users reported this was moderately likely or higher, though only 35% of users stated this was extremely likely.

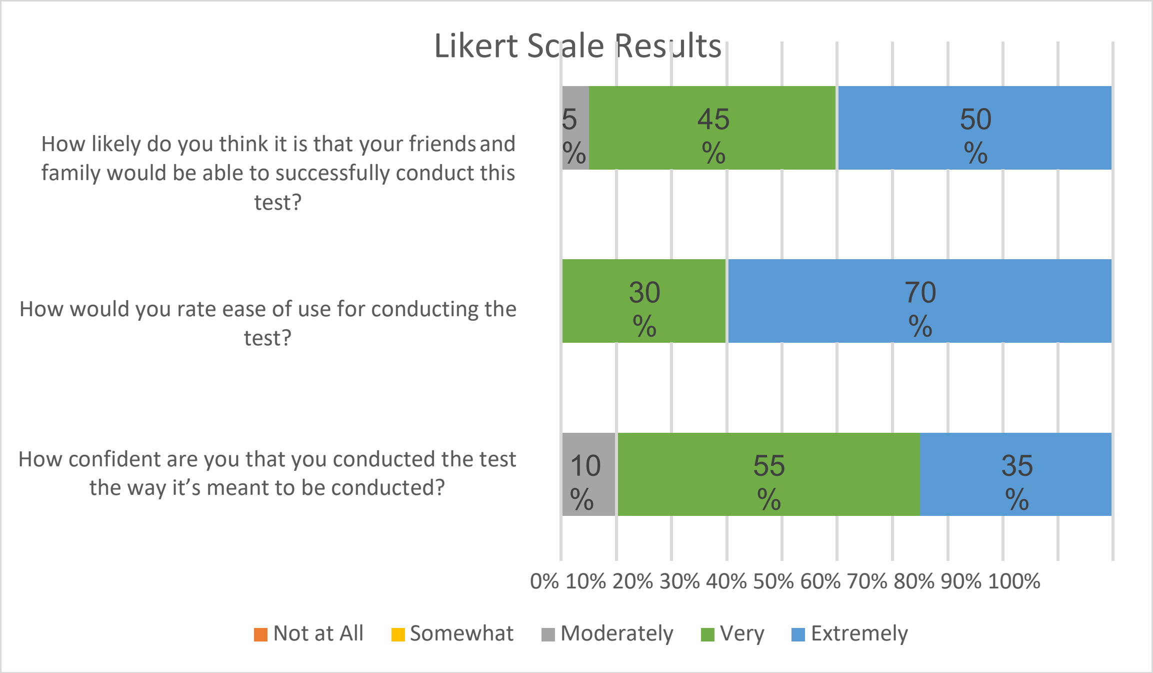

Figure 3. Likert Scale Results for self-administered tests.

4.3.2 Self-collected Samples

For novice users who self-collected their sample with verbal instructions from a practitioner, scale results (Figure 1; Self-collect data) skewed lower. When asked about the ease of use and confidence during use, some users reported "not at all" or "somewhat" responses, with only 29% extremely confident and 55% who found the sample extremely easy to collect. Users had more confidence in their friends and families, however, as all users reported it was at least somewhat likely their peers could successfully collect the sample.

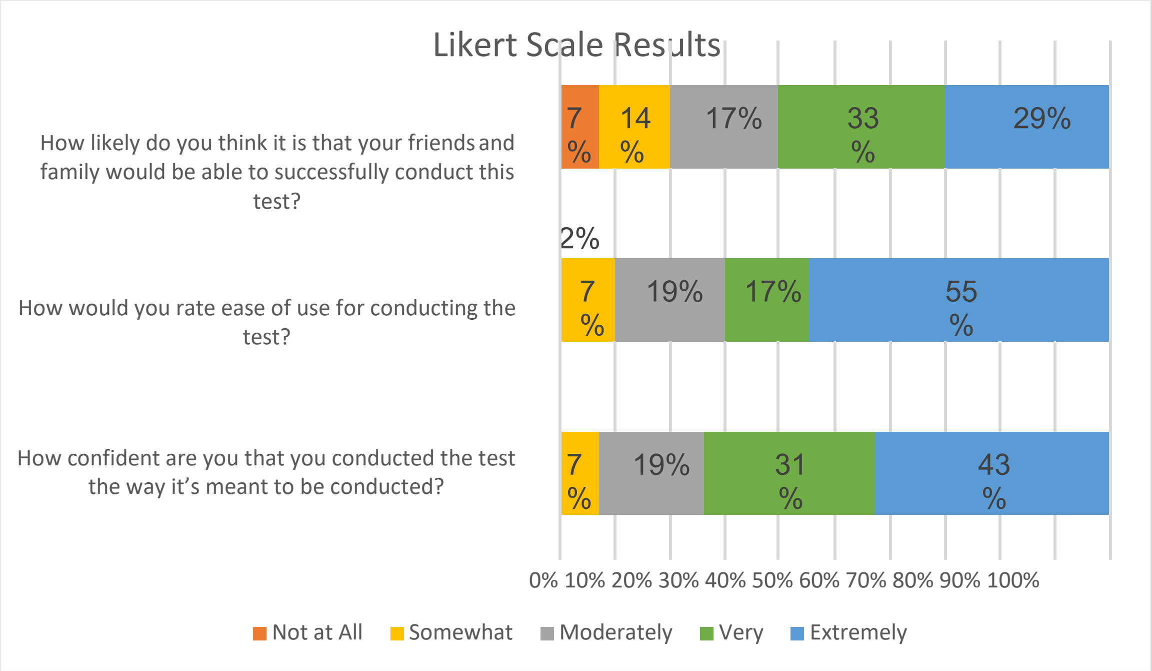

Figure 4. Likert Scale Results for self-collected samples.

5 Recommendations

Based on the various evaluations conducted and the feedback that was given by users, the following recommendations are suggested for increased usability.

1. Recommendation: Increase the font size for the procedure card to at least 12pt font. Expected Result of Implementation: This will make the instructions more accessible to users.

2. Recommendation: For instructions, use simple, direct language. Avoid medical jargon. Expected Result of Implementation: This will reduce user confusion.

3. Recommendation: Avoid using all caps in instructional materials. Consider using bolded or larger font and underlining as methods to create emphasis.
   Expected Result of Implementation: This will make the instructions more readable to the user.

4. Recommendation: Consider changing the images in the procedure card to more realistic drawings.
   Expected Result of Implementation: This will reduce the possibility of confusing users on how to correctly complete the protocol steps.

5. Recommendation: On the procedure card, highlight the "Important" section with larger text and other formatting strategies to draw the user's eye.
   Expected Result of Implementation: This will reduce the possibility of the user missing key information.

6. Recommendation: Print the product insert in color.
   Expected Result of Implementation: Because color indicates the validity of the test, colored instructions will more clearly communicate how to properly interpret the test results.

7. Recommendation: Provide a secondary indicator for invalid results.
   Expected Result of Implementation: Because color indicates the validity of the test, individuals unable to adequately interpret color will benefit from a secondary indicator.

8. Recommendation: Include sample collection instructions.
   Expected Result of Implementation: This will reduce the possibility of user error when collecting a sample.

9. Recommendation: Consider large tear away tabs on the device and swab packaging.
   Expected Result of Implementation: This will make the protocol easier for users with impaired dexterity.

10. Recommendation: Consider including pre-measured reagent packs or vials to eliminate the need to count drops into the device.
    Expected Result of Implementation: This will reduce the possibility of users over or under dispensing reagent and enhance the overall user experience.

11. Recommendation: On the device, consider labeling the swab insert holes to more easily differentiate the holes in the instructions, e.g., holes A and B or holes 1 and 2.
    Expected Result of Implementation: This will reduce confusion for the user while following the protocol.

12. Recommendation: Consider increasing the size of the reagent and swab insertion holes. Expected Result of Implementation: This will make the protocol easier for users with reduced dexterity.

6 F&F Usability Matrix

This homegrown rating scale is used as a means to quickly communicate overall usability of a system to various stakeholders, as well as a means to compare the usability of various systems. As indicated by color, the more usable a product is, the lower the usability score. The usability scale is scored on the following criteria:

Risk of design-induced errors

- Frequency and severity of potential errors
  1. Low risk of errors; unlikely to cause system failure

2. Moderate risk of errors; may cause system failure
3. High risk of failures; likely to cause system failure

Efficiency

- Number of steps required
- Amount of time required to complete steps
- Amount of resources expended to conduct test
- Including required attentiveness to IFU/order of steps
    1. High efficiency; unlikely to require large expenditure of resources to avoid system failure
    2. Moderate efficiency; may require moderate expenditure of resources to avoid system failure
    3. Low efficiency; system failure likely without large expenditure of resources

6.1 Point of Care

As the primary use case for the current design of the device, researchers evaluated the point of care usability by observing and interviewing intended end users and by conducting an expert evaluation.

As shown below in Figure 6, the usability score for the test is 2. Efficiency was scored 2, requiring moderate expenditure of resources from the user. Error was rated 1, low risk of failure. Moderate expenditure of resources is required given the number of steps, force required to administer drops, and the somewhat novel tasks associated with the procedure. The design of the device, such as the reservoir that is situated under both holes, allows for some flexibility in following the protocol.

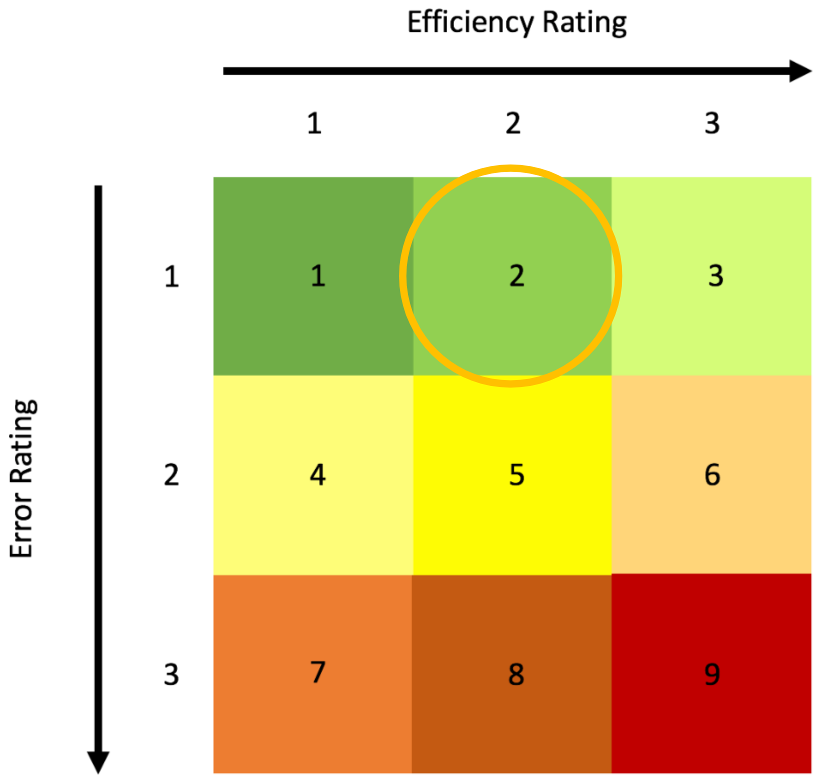

Figure 5: Usability- Point of care

6.2 Trained Layperson

While evaluating the device, the researchers considered the use case for a trained layperson, meaning a user who has been trained on the protocol, but does not have a medical background. This use case would be applicable for small-scale testing situations such as testing at work places, schools, or other similar scenarios.

Although not directly observed, User 2 fits this user description and used the device for two days. Researchers interviewed her about her experience with protocol. She found the process to be simple and noted that the only slight difficulty was administering the of drops into the device. Additionally, novice users observed at the drive-through testing site who conducted tests on more than one child experienced fewer errors and conducted tests more quickly on subsequent tests.

Given the results of the evaluation, observation, and interviews, the researchers found the device to be usable for the layperson use case. The F&F score for this use case was scored 2. Efficiency was scored 2, requiring moderate expenditure of resources from the user. Error was rated 1, low risk of failure. The tasks required for the procedure will likely be even more novel to an individual outside of the point of care setting, but with training, this will be abated.

Of the recommendations above, the greatest score impact would be made by including premeasured extraction reagent with lower force required to administer.

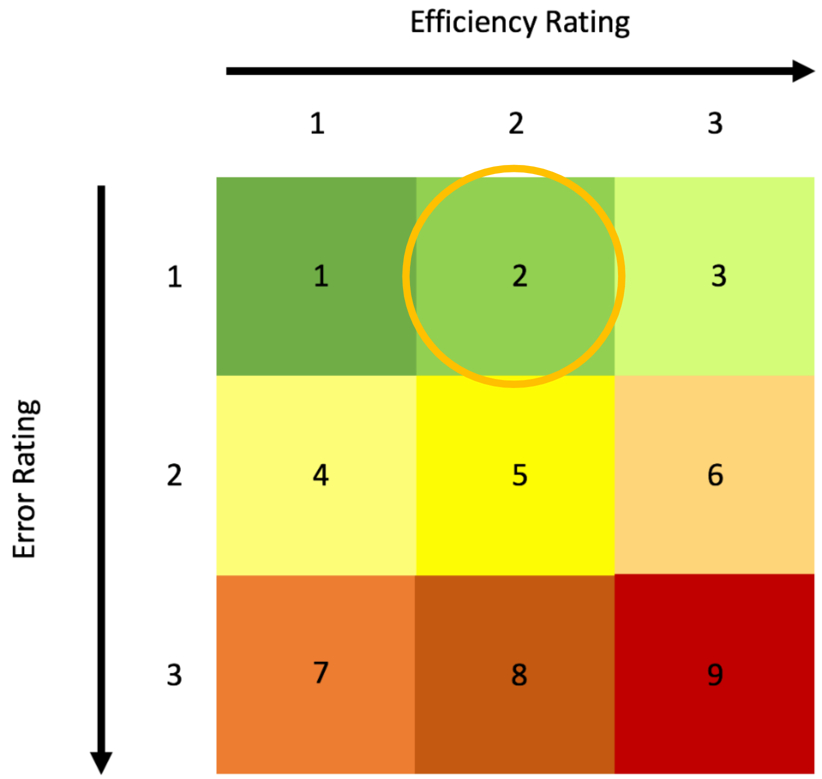

Figure 6: Usability- trained layperson

6.3 Home Use

While evaluating the device, the researchers considered the use case for home users. This use case would consist of users with no training and who would not perform a large volume of tests in any given period; i.e., individuals testing themselves or parents testing their child.

With some changes, the simple and streamlined design lends itself to a promising candidate for home use. Implementing pre-measured extraction buffer would also benefit this use case. The most critical improvements needed for this user population is improvements to the instructions for use, including instructions for sample collection, more realistic images, and implementation of simple, everyday language. This user group would also greatly benefit from additional training materials such an instructional video.

The F&F score for this use case is 5 (Figure 8). Efficiency was scored 2, requiring moderate expenditure of resources from the user. Error was rated 2, moderate risk of failure. Home users, who likely will find the procedure novel and may not benefit from the experience of running multiple tests, are more likely to encounter errors.

Of the recommendations above, the greatest score impact would be made by including premeasured reagents, increasing the reagent and swab holes in the device, and improving instructional materials.

"I think it could be sold to the public if you just add a packet of reagent."- User 1

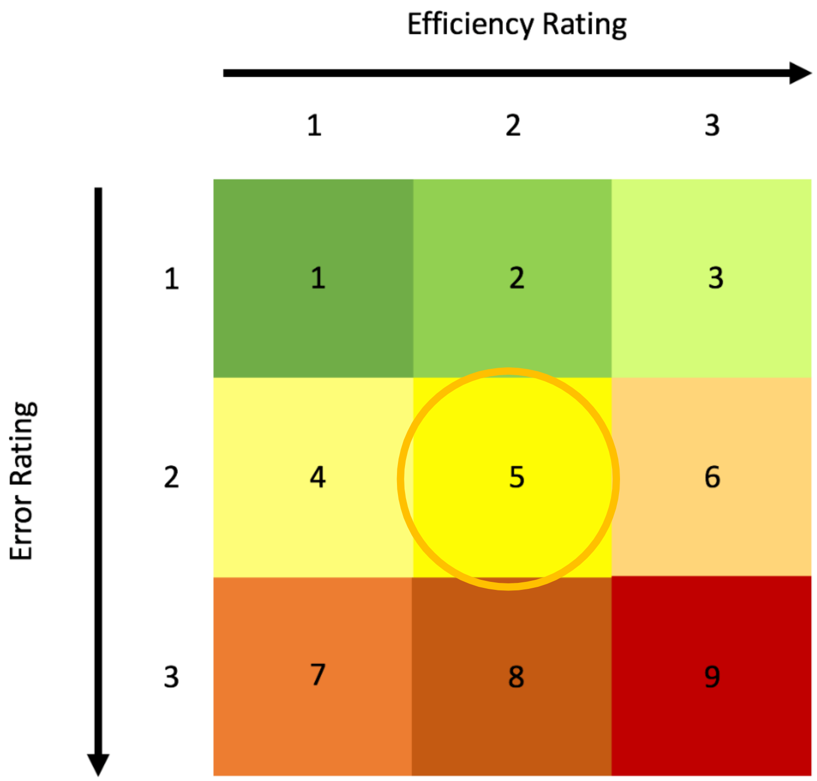

Figure 7: Usability- home use

7 References

Brooke, J. (1996). *SUS: a 'quick and dirty' usability scale.* In P.W.Jordan, B. Thomas, B.A. Weerdmeester, and I.L. McClelland (Eds.) Usability Evaluation in Industry (189-194). London: Taylor and Francis.

Mann, E., Baranack, A., & Fain, B. (2012). *Arthritis simulation gloves demonstration*. In 2012 CSUN Assistive Technology Conference proceedings.

O'Brien, Marita & Fausset, Cara & Weldon, Elizabeth & Harrington, Christina. (2015). *Using impairment simulation tools to demonstrate age-related challenges in everyday tasks and promote universal design*. Proceedings of the Human Factors and Ergonomics Society Annual Meeting. 58. 2402-2405. 10.1177/1541931214581600.

Sauro, J. & Lewis, J. R. (2016). *Quantifying the user experience: Practical statistics for user research.* Burlington, MA. Morgan-Kaufmann.

Shao, G. (2009, July). *System testing using use cases for an ER simulation model.* In North America Simulation Technology Conference 2009 proceedings.

Zimmerman Low Vision Simulation Kit, Pittsburgh, PA, USA. URL: http://www.lowvisionsimulationkit.com

8 Appendix A. Procedure Card.

Abbott

BinaxNOW™
COVID-19 Ag
CARD

Technical Support Advice Line
Further information can be obtained from your distributor, or by contacting
Technical Support on:
US +1 800 257 9525 ts.scr@abbott.com

PROCEDURE CARD

For Use Under an Emergency Use Authorization (EUA) Only.

The BinaxNOW COVID-19 Ag Card is a lateral flow immunoassay for the qualitative detection of the nucleocapsid protein antigen to SARS-CoV-2 directly from nasal swab specimens collected from individuals who are suspected of COVID-19 by their healthcare provider within seven days of the onset of symptoms.

IMPORTANT: **See Product Insert, including QC section, for complete use instructions, warnings, precautions and limitations.** False negative results may occur if specimens are tested past 1 hour of collection. Specimens should be tested as quickly as possible after specimen collection. **Open the test card just prior to use, lay it flat, and perform assay as follows.**

Part 1 - Sample Test Procedure

Patient Samples require 6 drops of Extraction Reagent.

1

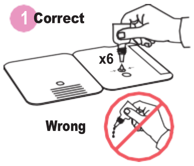

Hold Extraction Reagent bottle vertically. Hovering 1/2 inch above the TOP HOLE, **slowly add** 6 DROPS **to the** TOP HOLE **of the swab well.** DO NOT **touch the card with the dropper tip while dispensing.**

2

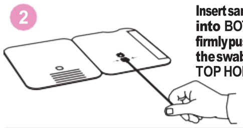

Insert sample or control swab into BOTTOM HOLE **and firmly push upwards so that the swab tip is visible in the** TOP HOLE.

3

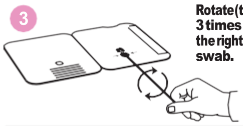

Rotate (twirl) swab shaft 3 times CLOCKWISE **(to the right). Do not remove swab.**

4

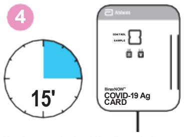

Peel off adhesive liner from the right edge of the test card. Close and securely seal the card. Read result in the window 15 minutes after closing the card. In order to ensure proper test performance, it is important to read the result promptly at 15 minutes, and not before. Results should not be read after 30 minutes.

Used test cards should be discarded as Biohazard waste according to Federal, State and local regulatory requirements.

Part 2 - Result Interpretation

A negative specimen **will give a single pink/purple colored Control Line in the top half of the window, indicating a negative result. This Control Line means that the detection part of the test was done correctly, but no COVID-19 antigen was detected.**

Negative results, from patients with symptom onset beyond seven days, should be treated as presumptive and confirmation with a molecular assay, if necessary, for patient management, may be performed.

Negative Result

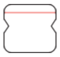

A positive specimen **will give two pink/purple colored lines. This means that COVID-19 antigen was detected. Specimens with low levels of antigen may give a faint Sample Line. Any visible pink/purple colored line is positive.**

Positive Result

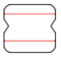

If no lines are seen, or if just the Sample Line is seen, the assay is invalid. Invalid tests should be repeated.

Invalid Result

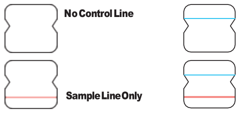

Procedure for External Quality Control Testing

External Controls require 8 drops of Extraction Reagent

1. **Hold Extraction Reagent bottle vertically. Hovering 1/2 inch above the** TOP HOLE, **slowly add** 8 DROPS **to the** TOP HOLE **of the swab well.** DO NOT **touch the card with the dropper tip while dispensing.**
2. **Follow Steps 2–4 of the Test Procedure shown.**

In the USA, this test has not been FDA cleared or approved; this test has been authorized by FDA under an EUA for use by authorized laboratories; use by laboratories certified under the CLIA, 42 U.S.C. §263a, that meet requirements to perform moderate, high or waived complexity tests. This test is authorized for use at the Point of Care (POC), i.e., in patient care settings operating under a CLIA Certificate of Waiver, Certificate of Compliance, or Certificate of Accreditation. This test has been authorized only for the detection of proteins from SARS-CoV-2, not for any other viruses or pathogens. In the USA, - this test is only authorized for the duration of the declaration that circumstances exist justifying the authorization of emergency use of *in vitro* diagnostics for detection and/or diagnosis of the virus that causes COVID-19 under Section 564(b)(1) of the Federal Food, Drug and Cosmetic Act, 21 U.S.C. § 360bbb-3(b)(1), unless the authorization is terminated or revoked sooner.

Abbott Diagnostics Scarborough, Inc.
10 Southgate Road
Scarborough, Maine 04074 USA
www.globalpointofcare.abbott

℞ Only
IVD

© 2020 Abbott. All rights reserved.
All trademarks referenced are trademarks of either the Abbott group of companies or their respective owners.

IN195001 Rev. 1 2020/08

Abbott
BinaxNOW
COVID-19 Ag

ProCard

Printed Colors

CMYK

PN: **IN195001**
Rev: **1**

Incoming Inspection Colors
(For Reference Only)
Colors below are not used for printing

PMS 2995U
Primary Blue

PMS 2995U
Primary Blue 70%

PMS 224 U
Magenta Pink

PMS 303 U
Dark Blue

PMS 185 U
Red

PMS 185 U
Red 70%

Size:
5.5" x8.0"

Date of Last Revision:
1.7 2020/08/25

References

1. Abbott Diagnostics. Abbott BinaxNOW COVID-19 Ag Card [Package Insert], version 1.6. 2020;
2. Harris PA, Taylor R, Thielke R, Payne J, Gonzalez N, Conde JG. Research electronic data capture (REDCap)--a metadata-driven methodology and workflow process for providing translational research informatics support. *J Biomed Inform*. Apr 2009;42(2):377-81. doi:10.1016/j.jbi.2008.08.010
3. Georgia Tech Launches HomeLab to Help Companies Evaluate In-Home Use of Emerging Health Technologies. June 12, 2012, 2012. Accessed January 12, 2021. https://rh.gatech.edu/news/134791/georgia-tech-launches-homelab-help-companies-evaluate-home-use-emerging-health
